# Posttraumatic Inflammation as a Key to Neuroregeneration after Traumatic Spinal Cord Injury

**DOI:** 10.3390/ijms16047900

**Published:** 2015-04-09

**Authors:** Arash Moghaddam, Christopher Child, Thomas Bruckner, Hans Jürgen Gerner, Volker Daniel, Bahram Biglari

**Affiliations:** 1 Heidelberg Trauma Research Group, Trauma and Reconstructive Surgery, Center for Orthopedics, Trauma Surgery and Spinal Cord Injury, Heidelberg University Hospital, Schlierbacher Landstraße 200a, D-69118 Heidelberg, Germany; E-Mail: hj.gerner@urz.uni-heidelberg.de; 2 Institute for Medical Biometry and Informatics, Heidelberg University Hospital, Im Neuenheimer Feld 305, D-69120 Heidelberg, Germany; E-Mail: bruckner@imbi.uni-heidelberg.de; 3 Department of Transplantation Immunology, Institute of Immunology, University of Heidelberg, Im Neuenheimer Feld 305, D-69120 Heidelberg, Germany; E-Mail: volker.daniel@med.uni-heidelberg.de; 4 Berufsgenossenschaftliche Unfallklinik Ludwigshafen, Department of Paraplegiology, Ludwig-Guttmann-Straße-13, D-67071 Ludwigshafen, Germany; E-Mail: bahram.biglari@bgu-ludwigshafen.de

**Keywords:** spinal cord injury, neuroregeneration, cytokines, inflammation, trauma

## Abstract

Pro- and anti-inflammatory cytokines might have a large impact on the secondary phase and on the neurological outcome of patients with acute spinal cord injury (SCI). We measured the serum levels of different cytokines (Interferon-γ, Tumor Necrosis Factor-α, Interleukin-1β, IL-6, IL-8, IL-10, and Vascular Endothelial Growth Factor) over a 12-week period in 40 acute traumatic SCI patients: at admission on average one hour after initial trauma; at four, nine, 12, and 24 h; Three, and seven days after admission; and two, four, eight, and twelve weeks after admission. This was done using a Luminex Performance Human High Sensitivity Cytokine Panel. SCI was classified using the American Spinal Injury Association (ASIA) Impairment Scale (AIS) at time of admission and after 12 weeks. TNFα, IL-1β, IL-6, IL-8, and IL-10 concentrations were significantly higher in patients without neurological remission and in patients with an initial AIS A (*p <* 0.05). This study shows significant differences in cytokine concentrations shown in traumatic SCI patients with different neurological impairments and within a 12-week period. IL-8 and IL-10 are potential peripheral markers for neurological remission and rehabilitation after traumatic SCI. Furthermore our cytokine expression pattern of the acute, subacute, and intermediate phase of SCI establishes a possible basis for future studies to develop standardized monitoring, prognostic, and tracking techniques.

## 1. Introduction

It is reported that the average annual incidence of SCI in the United States is 40 cases per million [1]. Damage to the spinal cord and the following consequences such as para- and tetraplegia have a devastating impact on the patient’s life. The injury is associated with dramatic medical, psychological, social, and economic consequences regarding the patient’s and his/her family’s life [2,3,4]. Depending on the extent and localization of the neurological damage, the deficits can range from incomplete sensory to a complete sensorimotor deficit. Secondary complications like chronic urinary tract infections, decubitus, cardiac, and vegetative dysregulation, as far as spinal shock, still threaten the patient’s life. Causal therapy for damaged structures still remains unfeasible [4]. Despite emergency rescues, advancing surgical techniques, and well-established physical therapies, chances of mobility and complete restitutio ad integrum continue to be limited [2]. There is an urgent need for both the scientific development and clinical validation of novel therapies for acute SCI [5].

The pathophysiology of SCI can be grouped into a primary and a secondary phase of injury. The primary phase is mainly marked by compression and energetic impact on the myelon. Possible consequences are axon disruption or bleeding in the surrounding tissue [6,7]. The primary phase triggers a series of mechanisms leading to secondary myelon damage within minutes after trauma. These effects can last for days after injury [8,9].

The neuroinflammatory component is of fundamental importance during the secondary phase of acute SCI. Although inflammatory processes remain physiological responses to tissue injuries, there is growing evidence of the negative effects of the neuroinflammatory processes during the acute and subacute phases after traumatic SCI. These processes are dependent on the secretion of pro- and anti-inflammatory cytokines [7,10,11,12,13]. Possible therapeutic targets can intervene especially in this stage. In our previous studies we analyzed the expression of soluble Cluster of Differentiation 95 Ligand, TNFα and IL-1β during the acute, subacute and intermediate phase of traumatic SCI in humans [14,15,16]. In our earlier collective, TNFα serum levels were significantly lower in patients with neurological remission nine hours after trauma. Furthermore, after a few weeks, TNFα levels showed a significant increase within the group with neurological remission [16].

In this study we examined multiple cytokines in a large patient collective (*n =* 40). These factors certainly play an important role, not only taken individually but also regarding the interactions between the cytokines. Therefore, the aims of the current study were the following:

(1) Is it possible to quantitatively measure the factors in the serum of traumatic SCI patients according to neurological impairment?
(2) Do the serum levels of patients with different neurological outcome differ from one another?

The current medicamentous, therapeutic possibilities after traumatic SCI are very limited. Therefore, results regarding this field of science can provide an important basis for future experimental and pharmacological studies.

## 2. Results

### 2.1. Patient Demographics

In the entire collective, there were 40 patients: 28 male and 12 female. The average age was 43.55 ± 20.80 years. Twenty-five injuries (62.5%) were due to falls, 13 (32.5%) traffic accidents, and two (5.0%) were from other causes. All patients had a Neurological Level of Injury (NLI) between C3 and L5. There were 17 cervical lesions (42.5%), 11 thoracic (27.5%), and 12 lumbar (30.0%). Initial AIS grades were as follows: 20 A injuries (50.0%), six B (15.0%), 13 C (32.5%), and one D (2.5%). Final AIS grades were as follows: 14 A (35.0%) injuries, four B (10.0%), seven C (17.5%), and 15 D (37.5%). Age, gender, etiology, AO-Classification, NLI and initial and final AIS grades for patients with and without AIS improvement are shown in Table 1.

**Table 1:** Demographic and clinical characteristics of subjects. Abbreviations: NLI = Neurological Level of Injury; AO = AO-Classification; AIS = American Spinal. Injury Association (ASIA) Impairment Scale. Age is expressed as mean years ± standard deviation. Neurological remission was defined as improvement in AIS. Three patients had posttraumatic spinal cord injury (SCI) without vertebral fractures. *p*-Values were analyzed with χ^2^-test (categorical data) and *t*-test (age) and show differences in distribution between G1 and G2.

Patients	N	Age (years)	Gender		Etiology			AO			NLI			Initial AIS				Final AIS			
			♀	♂	Fall	Traffic	Other	A	B	C	C	Th	L	A	B	C	D	A	B	C	D
All Patients	40	43.55 ± 20.80	12	28	25	13	2	19	13	5	17	11	12	20	6	13	1	14	4	7	15
Remission (G1)	23	43.26 ± 23.56	10	13	15	7	1	12	7	2	8	6	9	6	5	12	0	0	3	6	14
No Remission (G2)	17	43.94 ± 17.05	2	15	10	6	1	7	6	3	9	5	3	14	1	1	1	14	1	1	1
		*p* > 0.05	*p* < 0.05		*p* > 0.05			*p* > 0.05			*p* > 0.05			*p* < 0.05				*p* < 0.05			

There were 23 patients with AIS improvement and 17 with no improvement. Of the patients with improvement, the average age was 43.26 ± 23.56 years. There were 13 males (56.5%) and 10 females (43.5%). Injuries included 15 falls (65.2%), and seven traffic accidents (30.4%) and one other cause (4.3%). There were eight cervical lesions (34.8%), six thoracic (26.1%), and nine lumbar (39.1%). Initial AIS injuries included six A (26.1%) injuries, five B (21.7%), and 12 C (52.2%). Final AIS grades were as follows: three B (13.0%), six C (26.1%), and 14 D (60.9%).

Of the patients with no AIS improvement, the average age was 43.94 ± 17.05 years. There were two females (11.8%), and 15 males (88.2%). Injuries included 10 falls (58.8%), six traffic accidents (35.3%), and one other cause (5.9%). There were nine cervical lesions (52.9%), five thoracic (29.4%), and three lumbar (17.6%). Initial and final AIS grades included 14 A (82.4%), one B (5.9%), one C (5.9%), and one D (5.9%).

There was no significant difference in distribution of age, etiology, AO-classification, and NLI between patients with and without neurological remission (referred to as G1 and G2 in the following text, respectively). The percentage of men was significantly higher in G2 (*p <* 0.05). Furthermore the AIS grades at admission and discharge was significantly different in G1 and G2 (*p <* 0.05). Only six of 20 AIS A patients (30.0%) improved neurologically after 12 weeks but 17 of 20 AIS B–D patients (85.0%) had a better neurological outcome than before.

### 2.2. Analysis of Entire Patient Collective

**Figure 1:**
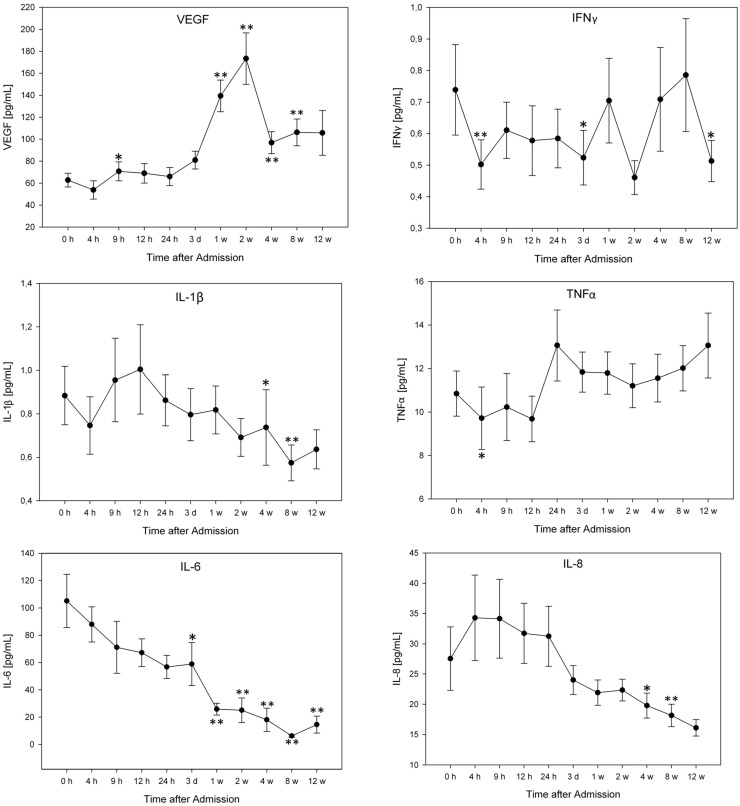

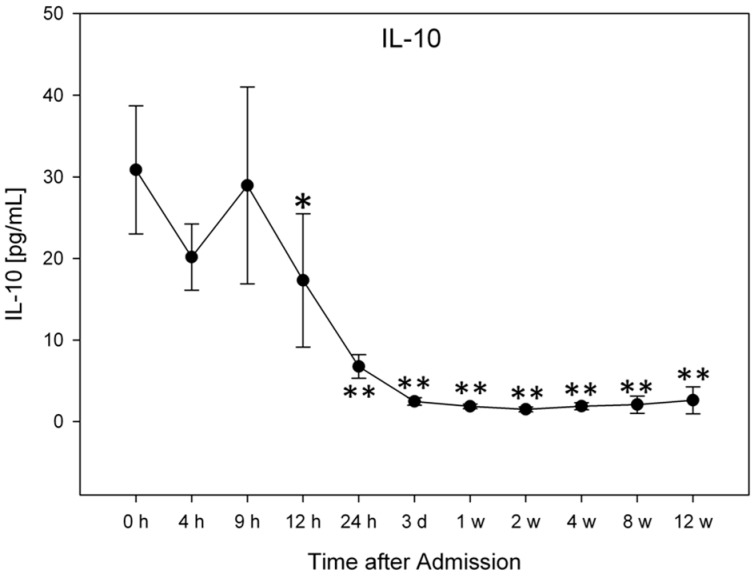
Serum levels of all traumatic SCI patients 12 weeks after admission, expressed as means ± standard error of the mean. The Wilcoxon signed-rank test assessed significant differences from the admission level (0 h) in each group, * *p <* 0.05, ** *p <* 0.01. Abbreviations: h = hours; d = day; w = week.

**Figure 2:**
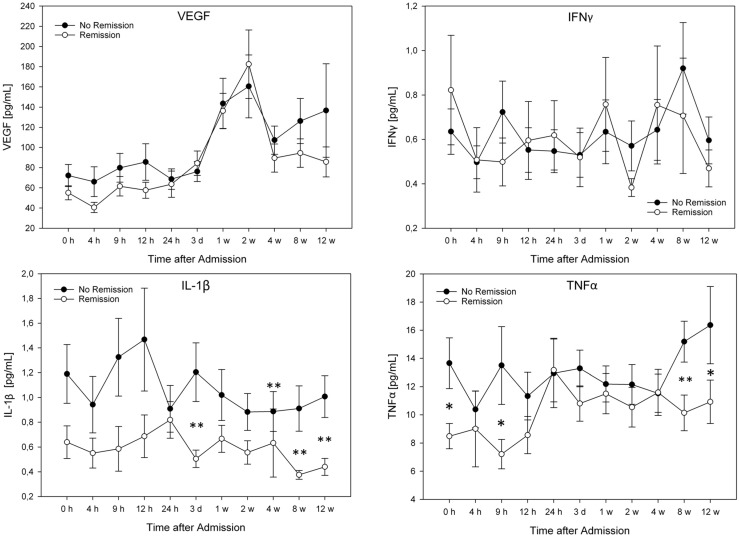

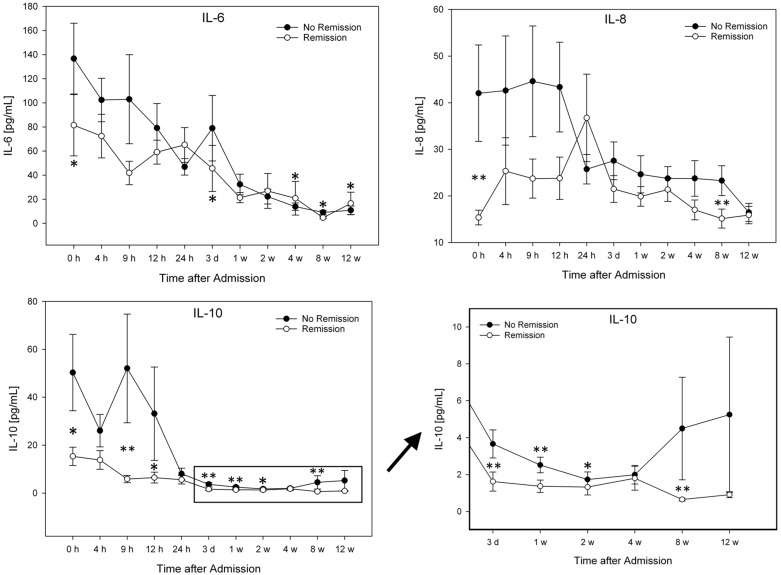
Serum level comparison of all patients with and without neurological remission (AIS improvement after 12 weeks). The Mann-Whitney-U-Test assessed significant differences between both groups at each particular time point, * *p <* 0.05, ** *p <* 0.01. Abbreviations: h = hours; d = day; w = week.

**Figure 3:**
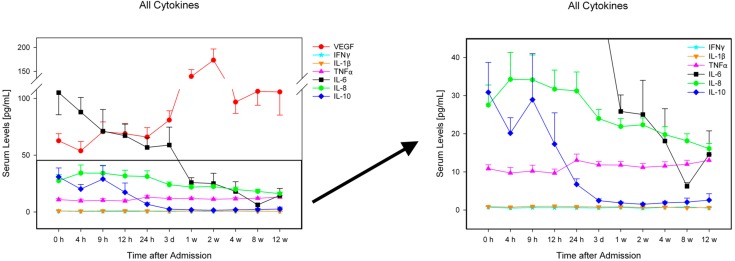
Serum levels of IFNγ, TNFα, IL-1β, IL-6, IL-8, IL-10, and VEGF in all traumatic SCI patients (*n =* 40) over a 12-week period, expressed as mean ± standard mean error. Abbreviations: h = hours; d = day; w = week.

For exact cytokine concentrations (pg/mL) and graphic comparison of the groups see Figure 1, Figure 2 and Figure 3. There were no significant differences in cytokine serum levels regarding gender, AO-classification, etiology, or NLI.

VEGF levels rose significantly nine hours after admission from 60.72 to 70.72 pg/mL (*p =* 0.02) and remained at this level until 24 h after admission. After one and two weeks, the concentration rose significantly (139.41 pg/mL, *p <* 0.0001; 173.38 pg/mL, *p <* 0.0001; Figure 1), decreased after four weeks (96.82 pg/mL), and remained at this level after 12 weeks (105.72 pg/mL). The values after four and eight weeks were still significantly higher than the initial value (*p <* 0.01; *p <* 0.001; Figure 1).

IFNγ significantly decreased to 0.502 pg/mL four hours after admission (*p =* 0.007), then increased after nine hours and remained at this level for 24 h. Three days after admission the concentration fell significantly to the initial value (*p =* 0.041).

IL-1β concentrations were significantly lower than the initial value after four and eight weeks (*p =* 0.019; *p =* 0.003; Figure 1).

The TNFα levels decreased significantly from 10.85 to 9.72 pg/mL (*p =* 0.045) four hours after admission.

During the period of 12 weeks, IL-6 levels continued to decrease. Three days after admission, concentrations fell significantly from 105.14 to 58.92 pg/mL (*p =* 0.013). All mean levels after one, two, four, eight, and 12 weeks were significantly lower than initial concentration (*p <* 0.0001; *p <* 0.001; *p <* 0.0001; *p <* 0.00001, *p <* 0.01; Figure 1). The mean IL-8 values were significantly lower than the initial values after eight and 12 weeks (*p =* 0.017; *p =* 0.003).

IL-10 levels were significantly lower after 12 h (*p =* 0.045), 24 h (*p =* 0.004), three days and one-12 weeks (*p <* 0.0001) than the initial level (Figure 1).

### 2.3. Comparison of Patients with Neurological Remission vs. without Neurological Remission (G1 vs. G2)

G1 IL-1β mean values were lower than G2 levels. Concentrations at admission, after three days, four, eight, and 12 weeks were significantly different (*p =* 0.031; *p =* 0.005; *p =* 0.004; *p =* 0.001, *p =* 0.002; Figure 2).

At admission, after nine hours, eight, and 12 weeks one could see significant differences in TNFα levels. Patients with neurological remission had significantly lower levels of TNFα than patients without neurological remission (*p =* 0.038; *p =* 0.013; *p =* 0.005; *p =* 0.021; Figure 2).

G1 and G2 followed a similar pattern as the entire patient collective regarding IL-6 levels. Nevertheless, G1 had lower IL-6 levels within the first 12 h after admission, significantly lower at admission (*p =* 0.033). In addition the values differed significantly after three days, and four, eight, and 12 weeks (*p =* 0.026; *p =* 0.043; *p =* 0.016; *p =* 0.029; Figure 2).

The IL-8 mean levels were clearly lower in G1 compared to G2. Especially the significantly lower initial value was noticeable (G2: 42.03 pg/mL–G1: 15.35 pg/mL; *p <* 0.001). G1 levels remained constantly lower than G2 levels, significantly lower after eight weeks (*p =* 0.004). Only after 24 h G2 levels were lower than G1 (Figure 2).

G1 IL-10 levels were lower than G2 levels at all time points, significantly lower at admission (*p =* 0.028), after nine hours (*p =* 0.004), 12 h (*p =* 0.037), three days (*p =* 0.003), one week (*p =* 0.005), two weeks (*p =* 0.043), and eight weeks (*p =* 0.002; Figure 2).

### 2.4. Comparison of Patients with AIS A and AIS B–D

We added a table with the data relating to the supplementary material.

#### 2.4.1. AIS A ↔ and AIS A ↑

Serum levels of TNFα, IL-1β, IL-6, IL-8, and IL-10 were significantly lower in patients with an initial AIS A who improved neurologically after 12 weeks (↑) compared with those who showed no improvement (↔).

#### 2.4.2. AIS B–D ↔ and AIS B–D ↑

Although patient numbers in both groups differed, we found a few significant differences in patients with an initial AIS B–D. Patients with neurological improvement had significantly lower concentrations than patients without neurological improvement in serum levels of TNFα, IL-6, IL-8 and IL-10.

#### 2.4.3. AIS A and AIS B–D

Comparing all the patients suffering an initial AIS A with patients suffering an initial AIS B–D (irrespective of their neurological outcome after 12 weeks), one could detect significant differences between both groups in serum levels of TNFα, IL-1β, IL-6, IL-8, and IL-10. Serum levels of AIS A patients were significantly higher. Each group had 20 patients.

#### 2.4.4. Receiver Operating Characteristic (ROC) Analysis

We analyzed all significant differences within the first 24 h between patients with and without neurological remission for a possible cutoff value (Table 2). The most applicable IL-8 and IL-10 values were 22.41 pg/mL with a sensitivity of 81.3% and a specificity of 84.2% (IL-8) and 4.83 pg/mL with a sensitivity of 92.3% and a specificity of 83.1% (IL-10).

**Table 2:** ROC-analysis of all significantly different time points in the first 24 h regarding neurological remission (Figure 2). Possible cutoff values with the best sensitivity/specificity were chosen. Values with an area under the curve of <0.75 and sensitivity or specificity of <0.6 were left out. * indicates a 95% confidence interval 0.711–0.993, *p* = 0.0004; † 95% confidence interval 0.664–0.999, *p* = 0.004.

Factor	Time after Admission	
	h0	h9
IL-8	22.41 pg/mL	
Sensitivity	0.813	
Specificity	0.842	
Area Under the Curve	0.852 *	
IL-10		4.83 pg/mL
Sensitivity		0.923
Specificity		0.692
Area Under the Curve		0.831 †

### 2.5. Data Analysis

Due to the partly non-normal distribution of the data, non-parametric tests were used to compare samples. Statistical analysis for two independent variables (cytokine concentrations of two different AIS groups or of two different demographics) was carried out with the Mann-Whitney-U-Test. Two dependent variables (cytokine concentrations within one group at different points of time) were compared with the Wilcoxon signed-rank test. Between group comparisons of categorical data were made with χ^2^-test. A ROC-analysis of all significantly different time points within the first 24 h was performed (Figure 2). The figures were illustrated as means and standard error of the mean. Statistical analyses were done with SPSS Software 22.0. Figures were created with Sigmaplot Software 11.0 (Systat Software, San Jose, CA, USA).

## 3. Material and Methods

Data were obtained from 40 patients (28 male, 12 female) with acute, traumatic SCI. All patients were admitted to the Berufsgenossenschaftliche Unfallklinik Ludwigshafen (BG Trauma Centre) between March 2010 and September 2014. The AIS grades were conducted in awake and responsive patients at time of admission and after 12 weeks according to the International Standards for Neurological Classification of SCI (ISNCSCI; Table 3) [17]. Demographic and clinical patient profiles are shown in Table 2. BG Ludwigshafen is a primary trauma center with its own helicopter. The included patients were admitted 1.24 ± 0.41 h after trauma.

Venous blood (7.5 mL Monovette, Sarstedt, Germany) was taken at 11 time points following injury: at admission, four, nine, 12, 24 h, three, seven days, two, four, eight, and 12 weeks after admission. After 20 min of coagulation, blood was centrifuged at 3000 rpm and stored at −80 °C until cytokine measurement.

The quantitative measurement of IFNγ, TNFα, IL-1β, IL-6, IL-8, IL-10, and VEGF from patient serum was done with the Luminex Performance Human High Sensitivity Cytokine Panel according to the manufacturer’s instructions. The kits were provided by R&D Systems (Minneapolis, MN, USA). The lab technician performing the test was blinded to all patients and clinical information. Lab work was done in the lab of the Heidelberg University Hospital.

**Table 3:** ASIA Impairment Scale (AIS) Grade and the functional impairment (Clinical State) due to Spinal Cord Injury (SCI) [18].

AIS Grade	Clinical State
A	Complete—No motor or sensory function is preserved in the sacral segments S4–S5
B	Incomplete—Sensory but not motor function is preserved below the NLI and includes the sacral segments S4–S5
C	Incomplete—Motor function is preserved below the NLI, and more than half of key muscles below the NLI have a muscle grade less than 3
D	Incomplete—Motor function is preserved below the NLI, and at least half of key muscles below the NLI have a muscle grade of 3 or more
E	Normal—Motor and sensory function is normal

This study was approved by the ethics committee of Rheinland Pfalz (Number: 837.266.09). All study participants signed an agreement to participate in this study. Patients with non-traumatic SCI, traumatic brain injury (TBI), severe abdominal trauma, traumatic amputations of the extremities, and comatose patients were excluded from the study. Patients received neither methylprednisolone sodium succinate (MPSS) nor nonsteroidal antiphlogistics.

Many of the measured concentrations of IFNγ and IL-1β were below detectable limits (IFNγ: <0.3052 pg/mL; IL-1β: <0.3418 pg/mL), but enough values differed to compare groups and to obtain significant differences. In the analysis we used the lower limit as fixed value regarding these factors. Therefore these values must be considered critically. Only a few measured IL-10 values were below detectable limits (<0.4639 pg/mL) and in these cases we applied the same technique.

## 4. Discussion

In this study, we observed that different expression patterns were apparent in the peripheral serum depending on neurological trauma. IL-8 and IL-10 had significant differences at admission (IL-8: *p =* 0.0004; IL-10: *p =* 0.028) and after 9 h (IL-10: *p =* 0.004) and might be peripheral biomarkers for neurological outcome and rehabilitation after traumatic SCI. In addition we were able to show a complete expression pattern over a 12-week period in the acute, subacute, and intermediate phase after traumatic SCI in humans. Although many studies compared the cerebrospinal fluid (CSF) and serum concentration after traumatic SCI in humans, this was the first study in which a large patient collective was examined prospectively over a long period of time, including clinical follow-up. Davies *et al.* [19] demonstrated significantly higher TNFα and IL-6 levels in the serum of traumatic SCI patients compared with a control group; however, the taking of blood samples was carried out at different time points after injury. Hayes *et al.* [20] pointed out that TNFα levels significantly increased in patients with chronic SCI. Apart from these studies, Kwon *et al.* [5] indicated a positive correlation between IL-6 and IL-8 CSF levels and the severity of neurological impairment. These results were affirmed in further animal studies [10,21]. Unfortunately, they did not have a follow-up and, therefore, could not study the regenerative aspect of different factors. Another study revealed a decrease in TNFα, IL-1β, IL-4, IL-6, and IL-12 in the spinal cord of rats suffering from SCI after implanting mesenchymal stem cells. Additionally, a functional SCI recovery triggered by the implantation was observed [22]. These results agree with our findings that decreased levels of TNFα, IL-1β, and IL-6 might be an indicator for a better neurological outcome after traumatic SCI. It still remains unclear whether cytokines have a neuro-regenerative or -degenerative function by recruiting immune cells [23,24,25]. Jiang *et al.* [26] examined the influence of acupuncture on the cytokine expression in rats and concluded that reduced levels of IL-1β, TNFα, and IL-6 might have a positive effect on neurological outcome. IL-8 might have positive as well as negative effects after traumatic SCI but few studies examined the IL-8 concentration after traumatic SCI. Nevertheless most of them report an upregulation and a severity-dependent release after SCI [5,27,28]. A few studies have shown the anti-inflammatory effect of IL-10 after SCI and concluded a neuroprotective effect, especially in the early stage of SCI [29,30,31]. Nevertheless, our results showed elevated IL-10 concentrations in patients without neurological improvement. This might be due to an injury-severity dependent release of IL-10. Many results differed in their opinion regarding the function of individual factors. IL-1β had been considered as having neuroprotective as well as neurodegenerating effects [32,33]. These discrepancies may be due to the recruitment of different cells by IL-1β, among other reasons [25]. Vidal *et al.* [34] examined the blockage of TNFα in the early phase of SCI in mice and could not detect a positive effect on the neurological outcome. TNFα as well as IL-1β are associated with neuropathic pain after SCI [35,36] but their role in the outcome after SCI still remains unclear. Pearse *et al.* [37] for example observed a Cyclic-Adenosine-Monophosphate (cAMP) dependent reduction of TNFα in mice, which could have been one of the factors leading to a functional recovery after SCI.

Most studies analyze the secondary phase of SCI by conducting animal models. In this connection, interesting findings have been obtained to develop possible therapies against the formation of glial scar tissue and to stimulate neuroregeneration. Nevertheless, clinical studies are of special interest due to the baseline differences between animal and human blood and CSF levels and the lack of transferability between the two study types [38]. Our results regarding the entire patient collective (*n =* 40) demonstrate interesting tendencies of serum levels (Figure 1, Figure 2 and Figure 3). VEGF serum levels are especially noticeable because of their similarity to serum levels after fractures [39]. Furthermore, VEGF and IFNγ levels indicate no difference between patients with and without neurological remission (Figure 2). The increased levels of VEGF and IFNγ could be an indication of a neurotrauma-unspecific release due to vertebral fractures in our patient pool.

Further research of therapeutic possibilities after traumatic SCI is of eminent importance. Current therapeutic options are very limited and thus there is a high need for improvement and optimization. Therefore, an early recognition of the severity and possible therapeutic strategy is essential. LaPar *et al.* [40] examined the cytokine release after mild and severe traumatic head injuries in humans. TNFα levels were significantly increased in severe head injuries, IL-6 and IFNγ in mild head injuries. These factors might be important prognostic markers after traumatic head injuries. We conducted a similar inquiry in our study regarding the prognosis of patients after traumatic SCI. The analysis of blood serum in contrast to CSF is particularly interesting. The measurement is peripheral and indeed further away from the affected area (central nervous system) but much more user-friendly in clinical daily routine and less invasive. The fact that we could measure discrepancies between different neurological impairments in the periphery might be due to the traumatic injury of the blood-brain barrier. As a result, otherwise neurospecific factors are able to enter the bloodstream through the damaged area. In our collective, patients without neurological remission had significantly higher levels of TNFα, IL-1β, IL-6, IL-8, and IL-10 than patients with remission (Figure 2). These results agree with our previous study with 23 patients. In the earlier study we could demonstrate significantly lower TNFα levels in patients without neurological remission nine hours after trauma [16]. The same results occur if one compares patients with similar impairments at admission (see supplementary data). All significant differences between AIS A and AIS B–D patients showed higher levels in patients without remission. This suggests that patients with a worse prognosis show increased cytokine levels. By comparing patients with similar neurological impairments at admission, the factor of increased cytokine levels due to the higher severity of injury (irrespective of the neurological damage) was eliminated and the observed differences in the results were more neurospecific.

Furthermore the comparison of AIS A with AIS B-D patients showed almost continuously higher average concentrations of TNFα, Il-1β, IL-6, IL-8, and IL-10 in patients with AIS A (Table 3). Since only 30% of the initial AIS A patients but nearly 85% of AIS B-D patients improved neurologically after 12 weeks, the results suggest prognostic features of the named serum factors (compare to Table 3). In the early acute phase within 12 h after admission, especially IL-8 and IL-10 stood out due to their considerably increased concentration in patients without remission.

Additionally we conducted a ROC analysis to evaluate a possible cutoff value for an early diagnosis of neurological outcome after traumatic SCI. Again IL-8 and IL-10 stood out in their results. Both factors show high sensitivity and specificity as possible biomarkers for early prognosis and therapy control. Nevertheless, the results have to be investigated more thoroughly in a larger collective and through further analyses.

## 5. Conclusions

In our study, different expression patterns of TNFα, IL-1β, IL-6, IL-8, and IL-10 were apparent in the peripheral serum depending on neurological trauma and remission. Especially IL-8 and IL-10 levels showed significant differences within the first 24 h after admission. This indicates that we are able to track processes after traumatic SCI in the peripheral serum, and that they are dependent on the neurological impairment and potential of remission. Furthermore our cytokine expression pattern of the acute, subacute, and intermediate phase of SCI establishes a possible basis for future studies to develop standardized monitoring, prognostic and tracking techniques.

## Supplementary Files

Supplementary File 1
